# Genipin cross-linked gelatin hydrogel for encapsulating wharton jelly mesenchymal stem cells and basic fibroblast growth factor delivery in vocal fold regeneration

**DOI:** 10.3389/fcell.2024.1489901

**Published:** 2024-12-05

**Authors:** Ng Wan-Chiew, Marina Mat Baki, Yogeswaran Lokanathan, Mohd Busra Fauzi, Mawaddah Azman

**Affiliations:** ^1^ Department of Otorhinolaryngology-Head and Neck Surgery, Faculty of Medicine, Universiti Kebangsaan Malaysia, Kuala Lumpur, Malaysia; ^2^ Department of Otorhinolaryngology-Head and Neck Surgery, Hospital Canselor Tuanku Muhriz, Universiti Kebangsaan Malaysia, Kuala Lumpur, Malaysia; ^3^ Department of Tissue Engineering and Regenerative Medicine, Faculty of Medicine, Universiti Kebangsaan Malaysia, Kuala Lumpur, Malaysia

**Keywords:** glottic insufficiency, vocal fold paresis/ paralysis, regeneration, hydrogel, gelatin

## Abstract

Approaches to regenerate vocal fold in glottic insufficiency remains to be a focus for exploration. This is attributed to the applications of cells or biological molecules alone result in fast degradation and inadequate for regeneration. Development of an injectable hydrogel for glottic insufficiency is challenging, as it needs to be non-cytotoxic, elastic yet possess good strength and easy to fabricate. This gap prompts us to study the feasibility of our genipin(gn)-crosslinked gelatin (G) hydrogel in encapsulating Wharton’s Jelly Mesenchymal Stem Cells (WJMSCs) and basic fibroblast growth factor (bFGF) WJMSCs with the aim to provide regeneration in glottic insufficiency. WJMSCs was encapsulated into two optimised formulations with the density of 2,000,000 cells/mL. The encapsulated cells were tested for its morphology, cell viability, proliferation and migration. Then, the incorporation of basic fibroblast growth factor (bFGF) was done into a final formulation and was tested for the cellular response and *in vitro* inflammation. 6G 0.4gn demonstrated better cell viability after *in vitro* culturing for 7 day. After incorporation of bFGF into cell-laden 6G 0.4gn, encapsulated WJMSCs showed to have improved viability and migration. The inflammatory profile of the hydrogel was imperceptible and was regarded as minimal or no pro- and anti-inflammation. Altogether, we have first formulated 6G 0.4gn which is suitable to encapsulate WJMSCs and incorporation of bFGF. Current study fulfils the market need in vocal fold regeneration, by suggesting its rejuvenating potential in glottic insufficiency, yet this combined formulation should be studied further to justify its translation to clinical setting.

## 1 Introduction

The negative consequences that arise from glottic insufficiency include difficulty of speaking, breathing or airway functionality. Researchers are continuously innovating new approach to mitigate the condition. A new treatment modality is recently revealed by (Hsieh et al., 2022) in clinical trial, namely APrevent^®^ Vocal-Implant-System (VOIS) provides a long-term augmentation and it is flexible for different size of glottic insufficiency closure. After implantation of the VOIS via medialization thyroplasty, saline is injected into the designated balloon through injection laryngoplasty to obtain the desired bulking volume. Despite the advantage being the adjustable bulking volume, this approach requires invasive approach and continuous monitoring for bulking volume as it does not help to rejuvenate the native tissue. Having said that, regenerative medicine is a potential approach to stimulate the regeneration of the native tissue in vocal fold. The current focus of research for vocal fold regeneration involves direct injection of autologous bone marrow mesenchymal stem cells (Clinical Trials.gov Number: NCT04290182), adipose derived mesenchymal stem cells (Clinical Trials.gov Number: NCT02904824), basic fibroblast growth factor (bFGF) (Abbaszadeh et al., 2020; Aboul-Soud et al., 2021; Huang et al., 2020; Rao et al., 2018), hepatocyte growth factor (HGF) (Kaboodkhani et al., 2021), platelet-rich plasma (PRP) (Clinical Trials.gov Number: NCT04839276, NCT03749863) and autologous fibroblast (Clinical Trials.gov Number: NCT02120781). However, these approaches have several limitations, including short augmentation and retention time, which result in minimal improvement. To address these limitations, some studies are focusing on developing biomaterials to augment the glottic gap, such as the use of “click chemistry”-based polyethylene glycol (PEG) for vocal fold injection, which was shown to augment the glottic gap of rabbits and maintain stability for up to 4 months (Kwon et al., 2021).

While biomaterials, cells, or biochemical factors can be applied alone, a combination of these approaches can improve regenerative efficiency (Bakhshandeh et al., 2017). Biomaterials can serve as a customised space for encapsulated cells to proliferate or secrete growth factors, as well as create a microenvironment for vascularisation, provide a localised effect and control biomolecules release for an extended period (Akter, 2016; Koh et al., 2020; Sabaghi et al., 2022). For vocal fold injection, hydrogel is practically applied due to its utmost properties to provide cell friendly environment, protect cell fate and can be injected via non-invasive procedure (Espona-Noguera et al., 2019). Along with that, drug delivery is also another superiority of hydrogel as it is capable in encapsulating and releasing protein molecules in a slow manner (Dreiss, 2020). Despite with its exceptional properties, the formulation of the hydrogel is needed to be fine-tuned based on the specific condition. The properties of the tuneable hydrogel are greatly affected by the type of biomaterials used, crosslinking method and crosslinking degree. One of the crosslinking methods include chemical crosslinking as it is efficient, durable and has adjustable mechanical properties (Echalier et al., 2019). However, chemically-crosslinked hydrogel potentially causes cytotoxic if the crosslinkers exceed the threshold. With that being said, our previous work had justified several formulations using genipin crosslinking in gelatin hydrogel with satisfied physical, chemical characteristics and cellular responses (Ng et al., 2023). Gelatin and genipin were chosen as they depicted low cytotoxicity and tuneable degradation by adjusting the crosslinking degree. These biomaterials were regarded to be superior compared to existing biomaterials for vocal fold injection such as calcium hydroxyapatite (CaHA), carboxymethylcellulose (CMC), and hyaluronic acid. These biomaterials were reported to have fast resorption and potential foreign body reaction (DeFatta et al., 2012; Wang et al., 2020; Wang and Carroll, 2021). In our previous study, these formulations were observed to have physical properties that might be suitable for vocal fold augmentation. These properties included gelation time within 20 min, low swelling ratio (less than 10%) and elastic modulus (between 2 and 10 kPa). Tissue engineering is defined by applying both biomaterials and cells together for therapeutic purpose (Raghav et al., 2022). Specifically, we tested WJMSCs in our previous study, with the final aim of encapsulating WJMSCs in the hydrogels. Hydrogels are known with high water content and elastic, making them to be a good candidate for cells encapsulation (Nicodemus and Bryant, 2008). Our hydrogels serve as potential materials to encapsulate cells as they showed microporous structures, hydrophilicity, presence of functional groups for cell-cell interactions and ability to maintain WJMSCs viability up to 7 days. Additionally, we further characterised WJMSCs to justify its functionality in encapsulating cells and biomolecules.

Mesenchymal Stem Cells (MSCs) from umbilical cord, or also named Wharton’s Jelly Mesenchymal Stem Cells (WJMSCs) is known to have better proliferation and plasticity than other MSCs from other sources, with additional advantages of easily obtained and minimal ethical issue (Corotchi et al., 2013). WJMSCs depict higher plasticity, angiogenesis and stemness among the stem cells (Christodoulou et al., 2013), providing better tissue regeneration and remodelling outcome via paracrine effect (Wu et al., 2022). The multipotency of WJMSCs might be beneficial for regeneration of vocal fold with complex microstructures. Currently, regeneration of WJMSCs was proven for cardiomyocytes (Cheng et al., 2022) and nerve in rat model (Gärtner et al., 2012). These components are abundant in vocal fold and their impairments might lead to glottic insufficiency. However, the evidence of WJMSCs in vocal fold regeneration is rarely reported, prompting us to investigate. On the other hand, bFGF applications in vocal fold regeneration have been applied commonly in clinical setting and demonstrated positive outcomes (Abbaszadeh et al., 2020; Aboul-Soud et al., 2021; Huang et al., 2020; Rao et al., 2018). This might due to the abundant fibroblasts in vocal fold, which then improve the extracellular matrix (ECM) production and native regeneration (Kishimoto et al., 2016). Integration of bFGF showed a positive impact on the cellular response of WJMSCs, by increasing the exosome secretion (Park et al., 2023). Moreover, the sustained release of bFGF in hydrogel via diffusion can be controlled by altering the pore size (Sheth et al., 2019). Sustained release of growth factors were shown to regenerate recurrent laryngeal nerve (RLN) in a rabbit model (Lasso et al., 2018). Therefore, we expect that the integration of WJMSCs and bFGF into the hydrogel provides a better outcome, as the native tissue might also appear as unfavourable microenvironment for the cells and growth factors to sustain longer. A biodegradable and non-cytotoxic hydrogel is very vital to act as a carrier, and slowly releases the cells and growth factors to the targeted area, while prolonging the regeneration effect (Guan et al., 2020). Meanwhile, the by-products release during degradation of hydrogel may result inflammation, which can worsen the situation. Acute inflammation in vocal fold needs to be managed carefully, as it can cause over swelling that will obstruct the airway causing life-threating event (Gupta & Mahajan, 2024). Therefore, we need to further investigate the feasibility of the proposed hydrogel for encapsulation as well as the inflammatory profile, as the crosslinking mechanism of genipin in the gelatin matrix may result cytotoxicity toward the encapsulated cells.

With the previous genipin-crosslinked gelatin hydrogel, we aim to propose a WJMSCs and bFGF encapsulation formulation that can provide a temporary bulking volume to the incomplete glottic, while gradually release the growth factor for a prolonged regenerative effect. Herein, current study serves as preliminary evidence to prove the encapsulation of WJMSCs and bFGF into our hydrogels, which is potential to apply in vocal fold regeneration.

## 2 Method

### 2.1 Fabrication of encapsulated WJMSCs in genipin-crosslinked gelatin hydrogel

Two hydrogels formulation (6G 0.4gn, 8G 0.4gn) were fabricated according to the previously established protocol (Ng et al., 2023). Both hydrogel formulations exhibited gelation within 20 min, biodegradable and biocompatible with WJMSCs after 7 days of *in vitro* culture suggesting their potential in encapsulation. Upon mixing of genipin granules (0.4% w/v) into the gelatin solution (6% or 8% w/v) at 50°C for 3 minutes, the liquid hydrogel was allowed to cool down in room temperature for 1 min. Then, cell density of 2,000,000 cells/mL was mixed with the liquid hydrogel and was fabricated in desired well plate.

### 2.2 Biocompatibility of WJMSCs in hydrogel

The cell viability assay of encapsulated WJMSCs was done using a LIVE/DEAD™ Viability/Cytotoxicity kit (Invitrogen, Waltham, MA, United States). The hydrogel with the encapsulated cell was rinsed with Dulbecco’s phosphate-buffered saline (DPBS). The staining solution included calcein-AM and ethidium homodimer-1 (ratio of 1:4), was applied to the hydrogel and stained for 30 min at 37°C. Greenly stained cells indicated viable cells while redly stained indicated dead cells. The image was obtained using a Nikon Eclipse Ti fluorescence microscope (Nikon, Tokyo, Japan) under a magnification of ×10. The viability of the cell was calculated via ImageJ software (NIH, Bethesda, MD, United States). The assessment was done on day one and seven after WJMSCs were encapsulated in the hydrogels.

### 2.3 Cell viability of WJMSCs in hydrogel

WJMSCs were encapsulated with an amount of 2,000,000 cells/mL in the hydrogel in a 96-well plate. After 24 h of culture, the viability of cells was examined by 3-(4,5-dimethylthiazol-2-yl)-2,5-diphenyl tetrazolium bromide (MTT) assay (Sigma-Aldrich, St. Louis, MO, United States). In general, the culture medium was removed and rinsed with DPBS. 90 ul of pure medium with 10 ul of 5 mg/mL MTT solution was put into each well, which yielded a diluted concentration of MTT assay (0.5 mg/mL). After incubation for 4 h at 37°C, 87.5 ul of the solution was removed and 100 ul of DMSO was added (Sigma-Aldrich, St. Louis, MO, United States) to solubilise the formazan. The final solution was read by spectrophotometer to examine its absorbance at 570 nm (BioTek, PowerWave XS, Highland Park, IL, United States). The cell viability determination using MTT assay was done on day one and seven of culturing respectively.

### 2.4 Morphology of WJMSCs in hydrogel (SEM)

A total of 2,000,000 cells/mL of WJMSCs was encapsulated into the hydrogel. Upon cell culturing for 2 days, the hydrogel was washed with DPBS gently. The hydrogel was fixed using 3% glutaraldehyde and then was serially dehydrated by ethanol. The hydrogel was lyophilized for 24 h and coated with a nanogold to analyse the cell structure through FESEM (Supra 55VP, Zeiss, Obenkochen, Germany). The image was viewed under ×400 magnification.

### 2.5 Immunocytochemistry (ICC) - Ki67 of WJMSCs in hydrogel

A total of 2,000,000 cells/mL of WJMSCs was encapsulated into the hydrogel in a 48-well plate. After 24 h, the hydrogel with WJMSCs encapsulation was rinsed with 500 μL of phosphate-buffered saline (PBS) thrice. Then, the hydrogel with WJMSCs encapsulation was fixed with 200 μL of 4% paraformaldehyde in a chill condition for 15 min. The washing of hydrogel with WJMSCs encapsulation was done with 500 μL PBS thrice at 5 minutes each in an incubator shaker. After washing, the hydrogel with WJMSCs encapsulation was treated with 200 μL of 0.5% triton-X (Sigma-Aldrich, St. Louis, MO, United States) for 20 min. The washing of hydrogel with WJMSCs encapsulation was done by 500 μL PBS three times at 5 minutes each in an incubator shaker before blocking steps with 10% of goat serum (Sigma-Aldrich, St. Louis, MO, United States) at 1 hour and 37°C. Primary antibody was prepared in 1% of goat serum. 200 μL of Ki67 recombinant rabbit monoclonal antibody (SP6) (1:1,000) (Invitrogen, Waltham, MA, United States) was added to the hydrogel with WJMSCs encapsulation for overnight incubation. Upon 20–22 h of incubation, the primary antibody was removed and cleaned with 500 μL PBS thrice at 5 minutes each. The goat anti-mouse IgG, Alexa Fluor^®^ 488 in 1% goat serum, dilution of (1:1,000) (Abcam, Waltham, MA, United States), was the secondary antibody and 200 μL of it was added into the hydrogel with WJMSCs encapsulation for incubation at 2 hours and 37°C. The secondary antibody was removed and cleaned with 500 μL PBS thrice at 5 minutes each. 1 μM of 4′,6-diamidino-2-phenylindole (DAPI) staining was prepared in PBS and 200 μL of the solution was added for incubation up to 20 min at room temperature. The final step included a washing procedure with 500 μL PBS three times at 5 minutes each. The hydrogel with WJMSCs encapsulation was immersed in PBS until the fluorescent image was obtained via a Nikon Eclipse Ti fluorescence microscope (Nikon, Tokyo, Japan) under the magnification of ×20.

### 2.6 Migration of WJMSCs in hydrogel

WJMSCs upon 70%–80% confluency were washed with 5 mL DPBS and then stained with 0.5 μg/mL of CellTracker™ Green CMFDA dye (Invitrogen, Waltham, MA, United States) in pure α-MEM up to 30 min staining at 37°C. After staining, the cells were cleaned with 5 mL DPBS. The cells were cultured in 10% human platelet lysate (HPL) α-MEM for at least 24 h before trypsinisation. Then, the stained cells were applied for cell encapsulation with a cell density of 2,000,000/mL in a 48-well plate. Another flask of WJMSCs was seeded on a 24-well plate with cell density of 8,000/cm^2^. After 24 h, the cells were cleaned with DPBS and their nuclei were stained with 2 μg/mL Hoescht dye (Invitrogen, Waltham, MA, United States), diluted in pure α-MEM up to 30 min at 37°C. The cells were cleaned and used for the subsequent procedure. On day one, the hydrogel with encapsulated WJMSCs was flipped onto the blue cells in a 24-well plate and viewed using a Nikon Eclipse Ti fluorescence microscope (Nikon, Tokyo, Japan) under a magnification of ×10. The same set of hydrogel was viewed under day three, and seven.

### 2.7 Cellular response of encapsulated WJMSCs and bFGF in hydrogel

Prior to this stage, only one final formulation was included, which was 6G 0.4gn as it supported cell growth over 7 days. In this stage of the study, two different sets were fabricated, which were hydrogel with WJMSCs encapsulation (2,000,000 cells/mL) only and encapsulation of WJMSCs with bFGF (0.1 μg/mL) in the hydrogel. This stage was to compare the effect of bFGF incorporation toward encapsulated WJMSCs in the hydrogel.

### 2.8 Cell viability of encapsulated WJMSCs in hydrogel with bFGF incorporation

Two different sets were fabricated, which were WJMSCs encapsulation (2,000,000 cells/mL) and WJMSCs with bFGF (0.1 μg/mL) encapsulation into the hydrogel in a 96-well plate. After 24 h of culture, the viability of cells was examined by MTT assay (Sigma-Aldrich, St. Louis, MO, United States). In general, the culture medium was removed and rinsed with DPBS. 90 ul of pure medium with 10 ul of 5 mg/mL MTT solution was put into each well, which yielded a diluted concentration of MTT assay (0.5 mg/mL). After incubation for 4 h at 37°C, 87.5 ul of the solution was removed and 100 ul of DMSO was added (Sigma-Aldrich, St. Louis, MO, United States) to solubilise the formazan. The final solution was read by spectrophotometer to examine its absorbance at 570 nm (BioTek, PowerWave XS, Highland Park, IL, United States). The cell viability determination using MTT assay was done on day one and seven of culturing respectively.

### 2.9 Immunocytochemistry (ICC) - Ki67 of encapsulated WJMSCs in hydrogel with bFGF incorporation

A total of 2,000,000 cells/mL of WJMSCs was encapsulated into the hydrogel in a 48-well plate. After 24 h, the hydrogel with WJMSCs encapsulation was rinsed with 500 μL of PBS thrice. Then, the hydrogel with WJMSCs encapsulation was fixed with 200 μL of 4% paraformaldehyde in a chill condition for 15 min. The washing of hydrogel with WJMSCs encapsulation was done with 500 μL PBS thrice at 5 minutes each in an incubator shaker. After washing, the hydrogel with WJMSCs encapsulation was treated with 200 μL of 0.5% triton-X (Sigma-Aldrich, St. Louis, MO, United States) for 20 min. The washing of hydrogel with WJMSCs encapsulation was done by 500 μL PBS three times at 5 minutes each in an incubator shaker before blocking steps with 10% of goat serum (Sigma-Aldrich, St. Louis, MO, United States) at 1 hour and 37°C. Primary antibody was prepared in 1% of goat serum. 200 μL of Ki67 recombinant rabbit monoclonal antibody (SP6) (1:1,000) (Invitrogen, Waltham, MA, United States) was added to the hydrogel with WJMSCs encapsulation for overnight incubation. Upon 20–22 h of incubation, the primary antibody was removed and cleaned with 500 μL PBS thrice at 5 minutes each. The goat anti-mouse IgG, Alexa Fluor^®^ 488 in 1% goat serum, dilution of (1:1,000) (Abcam, Waltham, MA, United States), was the secondary antibody and 200 μL of it was added into the hydrogel with WJMSCs encapsulation for incubation at 2 hours and 37 °C. The secondary antibody was removed and cleaned with 500 μL PBS thrice at 5 minutes each. 1 μM of 4′,6-diamidino-2-phenylindole (DAPI) staining was prepared in PBS and 200 μL of the solution was added for incubation up to 20 min at room temperature. The final step included a washing procedure with 500 μL PBS three times at 5 minutes each. The hydrogel with WJMSCs encapsulation was immersed in PBS until the fluorescent image was obtained via a Nikon Eclipse Ti fluorescence microscope (Nikon, Tokyo, Japan) under the magnification of ×20.

### 2.10 Migration of encapsulated WJMSCs in hydrogel with bFGF incorporation

WJMSCs upon 70%–80% confluency were washed with 5 mL DPBS and then stained with 0.5 μg/mL of CellTrackerTM Green CMFDA dye (Invitrogen, Waltham, MA, United States) in pure α-MEM up to 30 min staining at 37°C. After staining, the cells were cleaned with 5 mL DPBS. The cells were cultured in 10% HPL α-MEM for at least 24 h before trypsinisation. Then, the stained cells were applied for cell encapsulation with a cell density of 2,000,000/mL in a 48-well plate. Another flask of WJMSCs was seeded on a 24-well plate with cell density of 8,000/cm^2^. After 24 h, the cells were cleaned with DPBS and their nuclei were stained with 2 μg/mL Hoescht dye (Invitrogen, Waltham, MA, United States), diluted in pure α-MEM up to 30 min at 37°C. The cells were cleaned and used for the subsequent procedure. On day one, the hydrogel with encapsulated WJMSCs was flipped onto the blue cells in a 24-well plate and viewed using a Nikon Eclipse Ti fluorescence microscope (Nikon, Tokyo, Japan) under a magnification of ×10. The same set of hydrogel was viewed under day three, and seven.

### 2.11 Inflammatory properties of hydrogel

**FIGURE 1:**
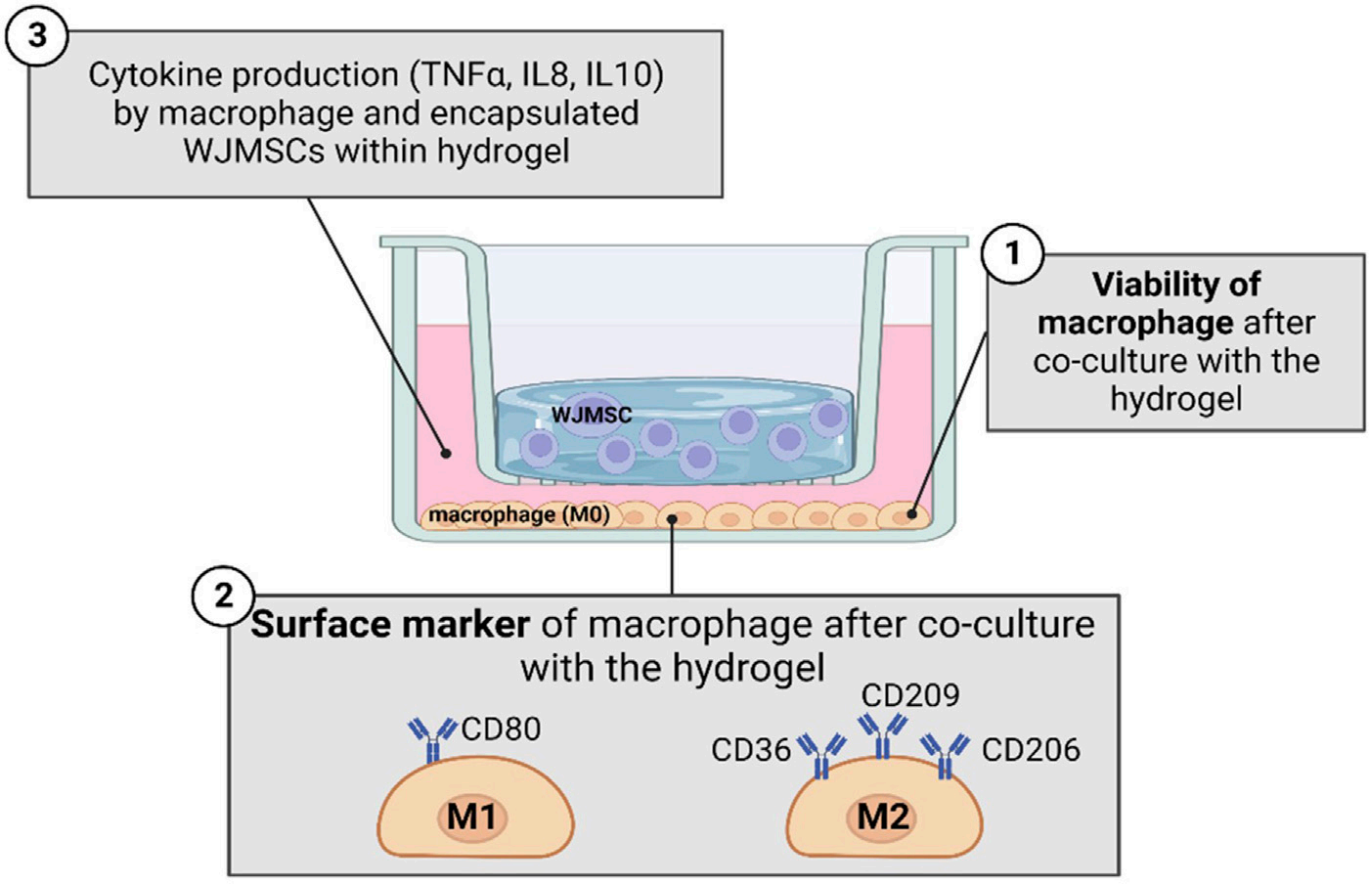
Schematic diagram in studying inflammation response of macrophage with co-culture of hydrogel. The figure was created using Biorender.com, the date of access 08 January 2023.

The cell line of THP-1 (ATCC, Manassas, Virginia, US) was revived from cryopreservation and was cultured with Roswell Park Memorial Institute (RPMI)-1,640 (Gibco, CA, United States) supplemented with 5% in-house HPL. After 3 days of culturing, total of 3,000,000 THP-1 cells were seeded into each 6-well plate for macrophage differentiation purpose. The differentiation medium was prepared with final concentration of 10 ng/mL of phorbol-12-myristate-13-acetate (PMA). After 24 h of differentiation, macrophage was observed to attach on the well plate and was co-cultured with hydrogel. Inflammatory properties of hydrogel were compared between five sets co-cultured with macrophages, which were i) hydrogel alone, ii) hydrogel with WJMSCs encapsulation only, iii) hydrogel with WJMSCs and bFGF encapsulation, iv) WJMSCs only and v) macrophage only (control). After co-cultured for 72 h, the subsequent tests were conducted. The experimental design was shown in Figure 1.

### 2.12 Biocompatibility of macrophage when co-cultured with hydrogel

After co-culture of the macrophage with hydrogel for 72 h, the macrophage was washed with 1 mL DPBS. Then, staining solution included calcein-AM and ethidium homodimer-1 (ratio of 1:4) in LIVE/DEAD™ Viability/Cytotoxicity kit (Invitrogen, Waltham, MA, United States) was prepared. 750 μL of the staining solution was put to the macrophage and stained for 30 min. The greenly-stained cells exhibited live cells while redly-stained cells exhibited dead cells. The image was obtained via a Nikon Eclipse Ti fluorescence microscope (Nikon, Tokyo, Japan) under a magnification of ×20.

### 2.13 Surface marker expression of macrophage when co-cultured with hydrogel

After co-culture of macrophage with hydrogel for 72 h, the macrophage was washed with DPBS and trypsinised. Each group was counted to have approximately 1 million cells and were stained with FITC anti-mouse CD36 antibody, PerCp/Cyanine 5.5 anti-mouse CD80 antibody, PE anti-mouse CD206 antibody, and APC anti-mouse CD209 (Biolegend, San Diego, CA, US). The stained macrophage was then read through flow cytometry (BD Sciences, Franklin Lakes, NJ, US).

### 2.14 Cytokine release of macrophage when co-cultured with hydrogel

After co-culture of macrophage with hydrogel for 72 h, the medium was collected. The cytokine release, namely tumor necrosis factor-alpha (TNF-α), interleukin (IL)-8, and IL-10 were quantified by ELISA test kit (Elabscience, Wuhan, China). The undiluted sample was used in the determination of TNF-α and IL-10 while a dilution factor of 1:50 was prepared for the determination of IL8. 100 μL of the sample was put into the well and incubated for 90 min at 37 °C. The sample solution was replaced by 100 μL of biotinylated detection antibody and underwent 1 h incubation at 37 °C. Then, three times washing were done by using 350 μL of wash buffer and then 100 μL of horseradish peroxidase (HRP) conjugate working solution was put for 30 min incubation at 37 °C. Five times washing were done by using 350 μL and then 90 μL substrate reagent was put for 15 min incubation at 37 °C. The last step included addition of 50 μL stop solution and the wells were viewed under 450 nm absorbance through a spectrophotometer (BioTek, PowerWave XS, Highland Park, IL, United States). A standard graph was plotted to determine the concentration of the tumor necrosis factor-alpha (TNF-α), interleukin (IL)-8, and IL-10.

### 2.15 Statistical analysis

Each hydrogel group was tested in triplicate and was repeated with thrice to ensure the duplicability. The data was interpreted as mean ± standard deviation and the data analysis was done by using GraphPad Prism version 8.0 (GraphPad Software, Inc., San Diego, CA, United States). Statistical analysis between the control group and treatment groups was conducted by one-way, two-way analysis of variance (ANOVA), and paired t-test. In certain tests, 0.1% genipin was used as a control group because previous studies proved that 0.1% was not cytotoxic (Zawani et al., 2022).

## 3 Result

### 3.1 Cellular response of encapsulated WJMSCs in hydrogel

#### 3.1.1 Morphology of encapsulated WJMSCs in hydrogel

**FIGURE 2:**
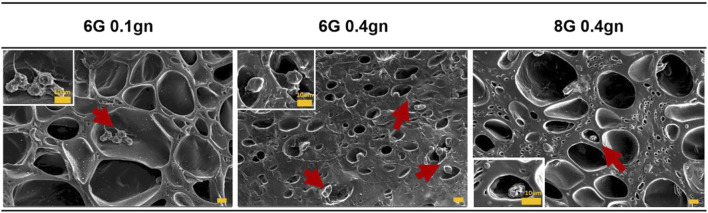
Morphology of WJMSCs after encapsulated in genipin-crosslinked gelatin hydrogels. The cells were viewed under ×400 magnification and each scale bar represents 10 μm.

After 2 days of culturing, WJMSCs that were encapsulated in genipin-crosslinked gelatin hydrogels were observed to remain in spherical shaped (Figure 2). The cells appeared to be staying in the pores of the hydrogels. However, WJMSCs in 6G 0.1gn showed pseudopodia, which were not observed in 6G 0.4gn and 8G 0.4gn.

#### 3.1.2 Cell viability of encapsulated WJMSCs in hydrogel

**FIGURE 3:**
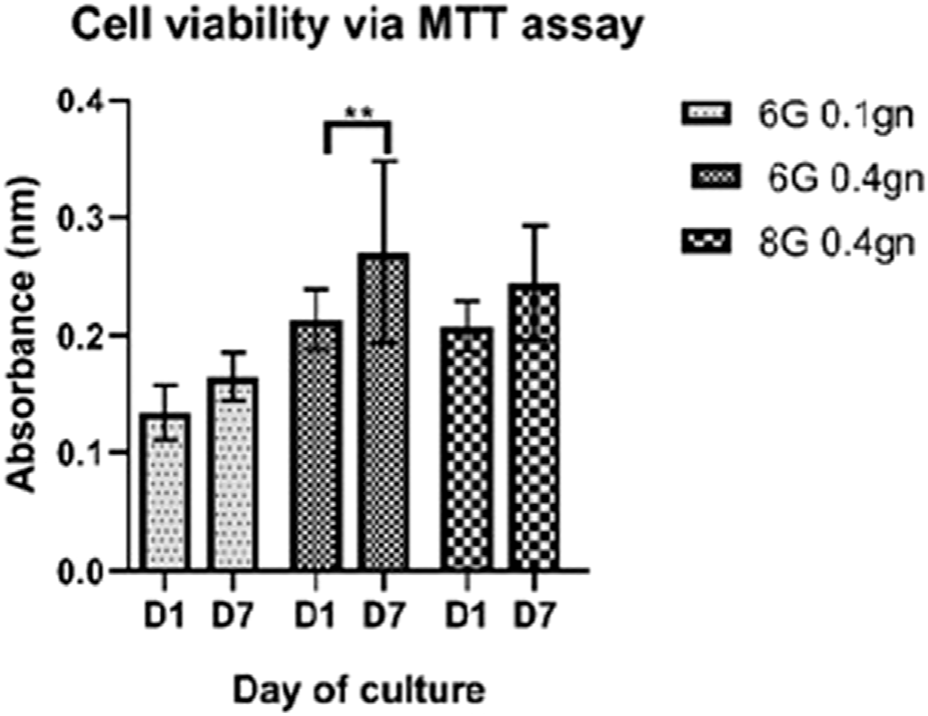
Viability of encapsulated WJMSCs from day one to seven of culturing in genipin-crosslinked gelatin hydrogels. ** indicates p < 0.01.

The viability of WJMSCs after encapsulation for one and 7 days were determined using MTT assay, which reflects the mitochondrial activity of viable cells. The absorbance readings showed that only the 6G 0.4gn group exhibited a significant increase in cell viability from day one to day seven (Figure 3). The other hydrogel groups had only marginal increases in viability, including 6G 0.1gn and 8G 0.4gn.

#### 3.1.3 Biocompatibility of encapsulated WJMSCs in hydrogel

**FIGURE 4:**
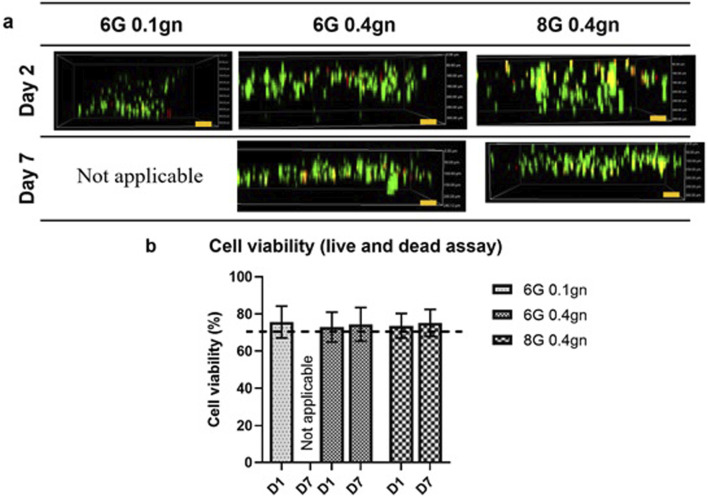
Cellular response via LIVE/DEAD™ Viability/Cytotoxicity assay. **(A)** Encapsulated WJMSCs and **(B)** the cell viability of encapsulated WJMSCs in genipin-crosslinked gelatin hydrogel. The cells were viewed under ×10 magnification and each scale bar represents 100 μm.

The viability of WJMSCs encapsulated within the hydrogels was found to be lower compared to the seeding method as reported previously. Specifically, the viability of encapsulated WJMSCs in 6G 0.4gn and 8G 0.4gn on day one was comparable to that of 6G 0.1gn (Figure 4B). However, on day seven, degradation of 6G 0.1gn resulted in an inability to conduct the test. Nonetheless, the viability of cells in 6G 0.4gn and 8G 0.4gn was maintained at minimum of 70% (Figure 4).

#### 3.1.4 Immunocytochemistry (ICC) – Ki67 of encapsulated WJMSCs in genipin-crosslinked gelatin hydrogel

**FIGURE 5:**
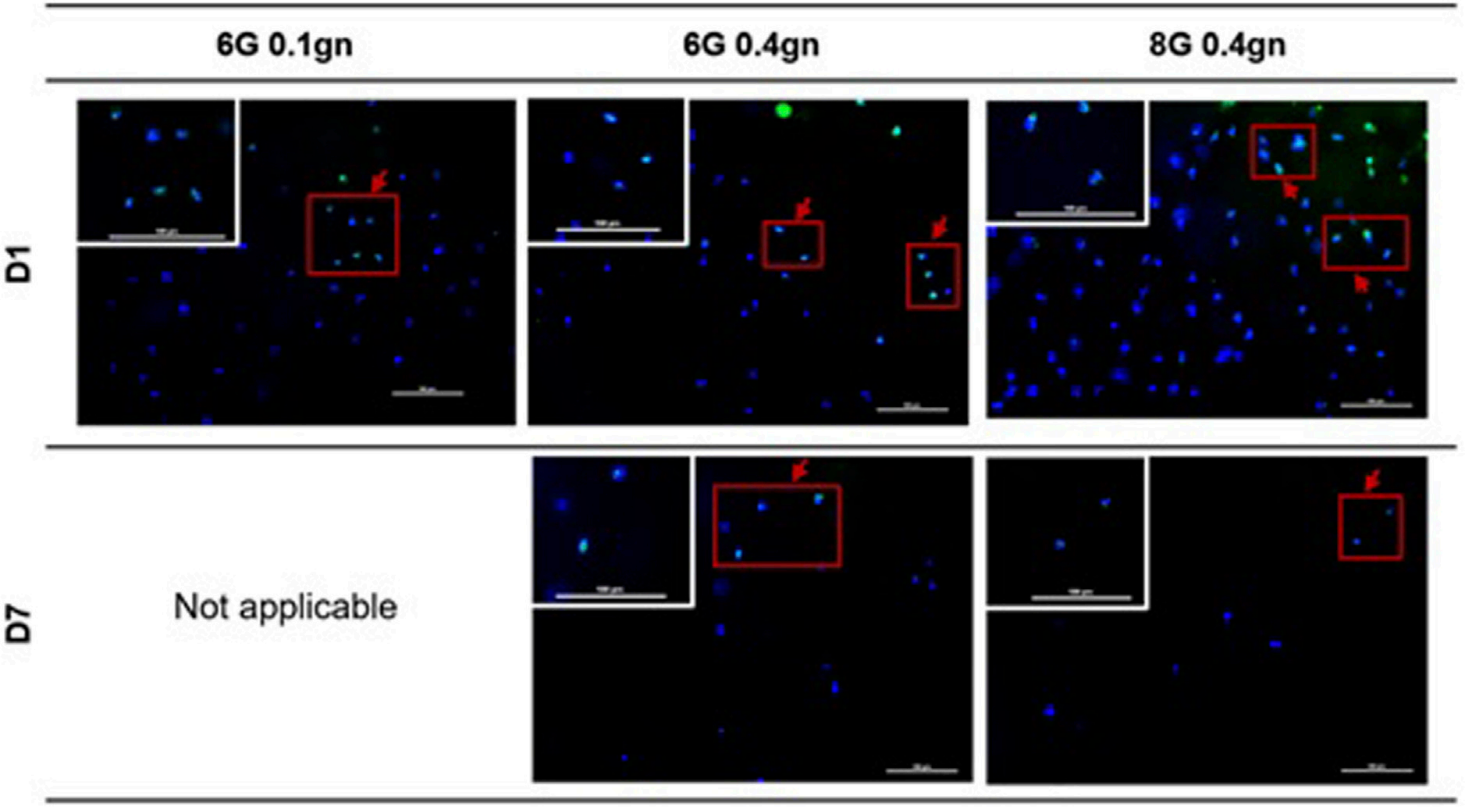
Proliferation markers of encapsulation WJMSCs in genipin-crosslinked gelatin hydrogel. The cells were viewed under ×20 magnification and each scale bar represents 100 μm.

Ki67 is commonly used to detect proliferating cells. All of the encapsulated WJMSCs in hydrogel sets showed to express the ki67 marker via ICC assay. The proliferation marker was maintained until day seven as according to our test (Figure 5).

#### 3.1.5 Migration of encapsulated WJMSCs in hydrogel

**FIGURE 6:**
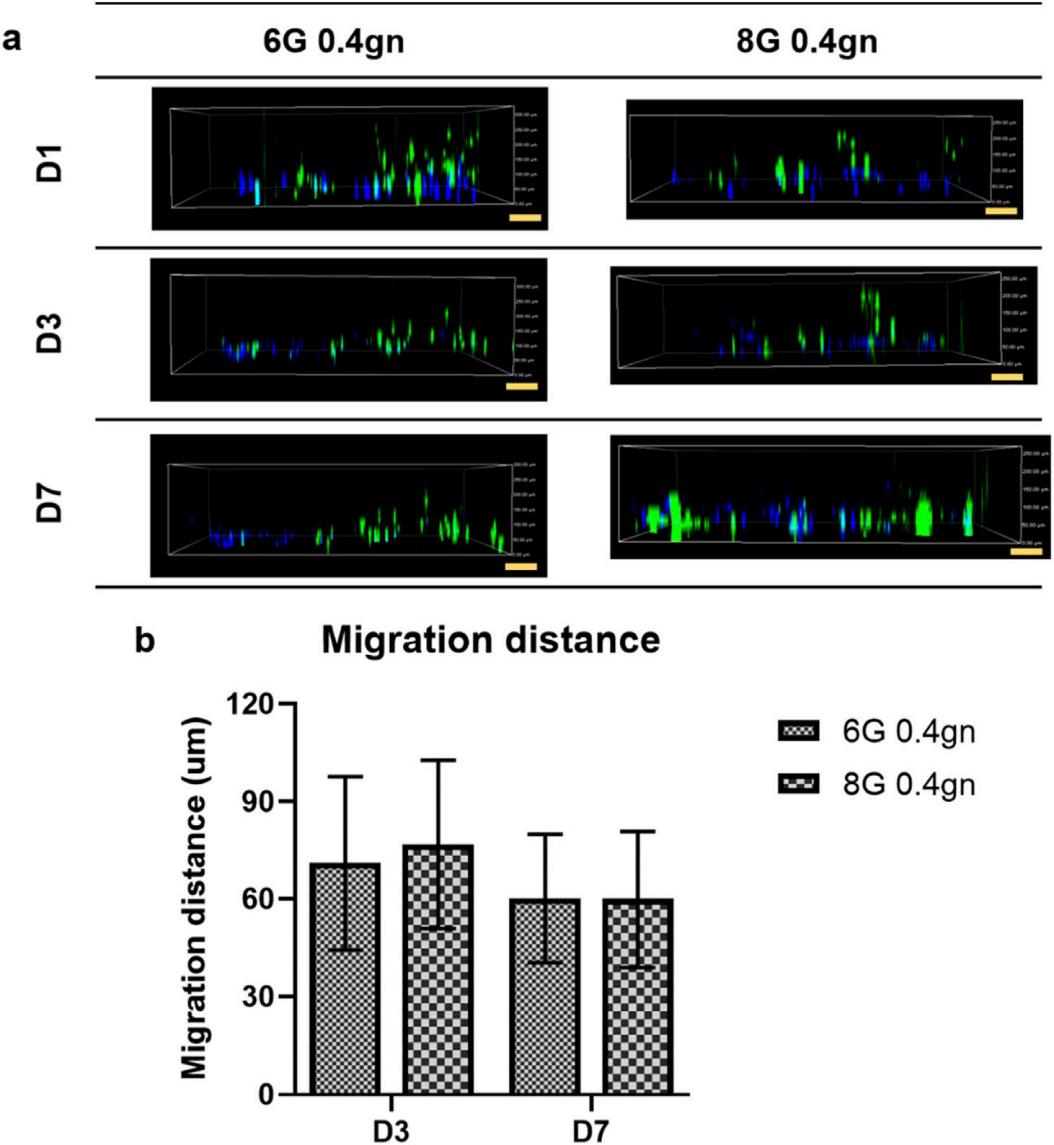
Migration of WJMSCs in genipin-crosslinked gelatin hydrogels up to 7 days. The cells were viewed under ×10 magnification and each scale bar represents 100 μm. The 6G 0.1gn group was not included because the hydrogel completely disintegrated starting from day 2. Therefore, the migration of the cells were not able to be observed and quantified. **(A)** 2D view of encapsulated WJMSCs in hydrogel and **(B)** calculated migration distance of encapsulated WJMSCs as compared to previous distance (D1-D3, D3-D7).

The genipin-crosslinked gelatin hydrogels were able to create a suitable homing environment that facilitated the migration of WJMSCs (as shown in Figure 6). The migration distance of WJMSCs was measured using the ImageJ software. The encapsulated WJMSCs in both 6G 0.4gn and 8G 0.4gn hydrogels were observed to migrate over time, with no significant difference between the two groups.

The results of the encapsulation study showed that both hydrogels, 6G 0.4gn and 8G 0.4gn, supported the viability, proliferation, and migration of WJMSCs. However, only 6G 0.4gn was able to significantly increase cell viability after 7 days of culturing *in vitro*. Therefore, 6G 0.4gn was selected as the final hydrogel formulation for the incorporation of bFGF in the subsequent experiments.

#### 3.1.6 Cell viability of encapsulated WJMSCs in hydrogel bFGF incorporation

**FIGURE 7:**
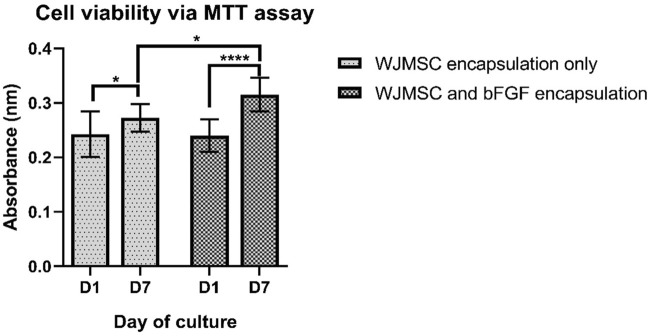
Cell viability of WJMSCs after encapsulated in genipin-crosslinked gelatin with or without the bFGF incorporation up to 7 days. * indicates p < 0.05, **** indicates p < 0.0001.

The MTT assay results revealed an increase in absorbance readings for both cell viability with or without bFGF incorporation at day seven, indicating an increase in metabolic activity of the cells (Figure 7), while cell and bFGF encapsulation showed an even more significant increase. Interestingly, WJMSCs in hydrogel incorporated with bFGF exhibited higher cell viability compared to those without bFGF incorporation at day seven (p < 0.05), indicating that bFGF incorporation may assist in the survival of WJMSCs in genipin-crosslinked gelatin hydrogel.

#### 3.1.7 Immunocytochemistry (ICC) – Ki67 of encapsulated WJMSCs in genipin-crosslinked gelatin hydrogel bFGF incorporation

**FIGURE 8:**
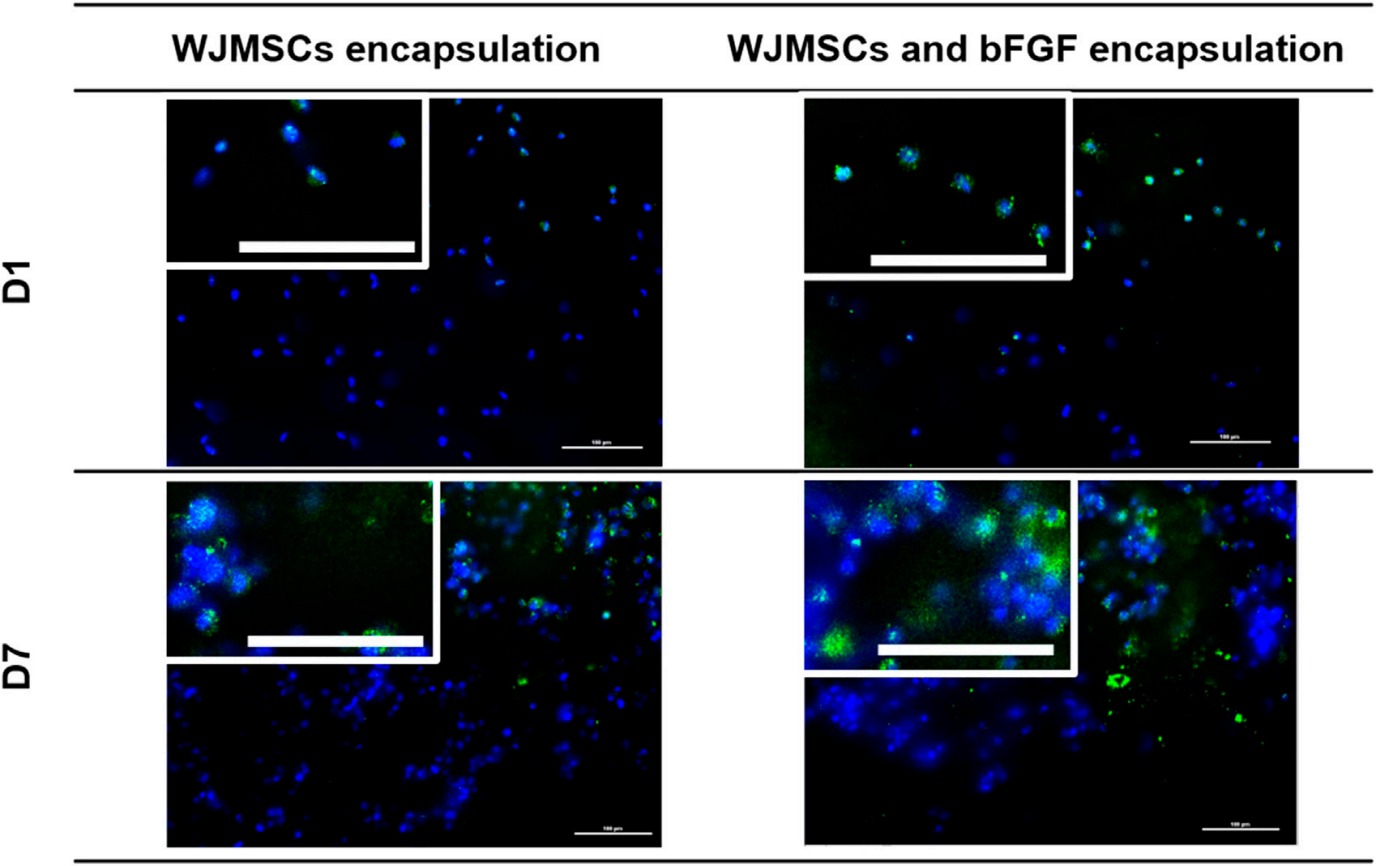
Proliferation markers of encapsulation WJMSCs in genipin-crosslinked gelatin hydrogel with or without bFGF incorporation. The cells were viewed under ×20 magnification and each scale bar represents 100 μm. A zoomed version of the cells were showed at the upper left of the image.

The ki67 staining showed a more prominent signal in WJMSCs encapsulated in bFGF-incorporated hydrogel (Figure 8). This qualitative result is consistent with the previous finding of increased cell viability through MTT assay, suggesting that bFGF promotes cell proliferation.

#### 3.1.8 Migration of encapsulated WJMSCs in genipin-crosslinked gelatin hydrogel with bFGF incorporation

**FIGURE 9:**
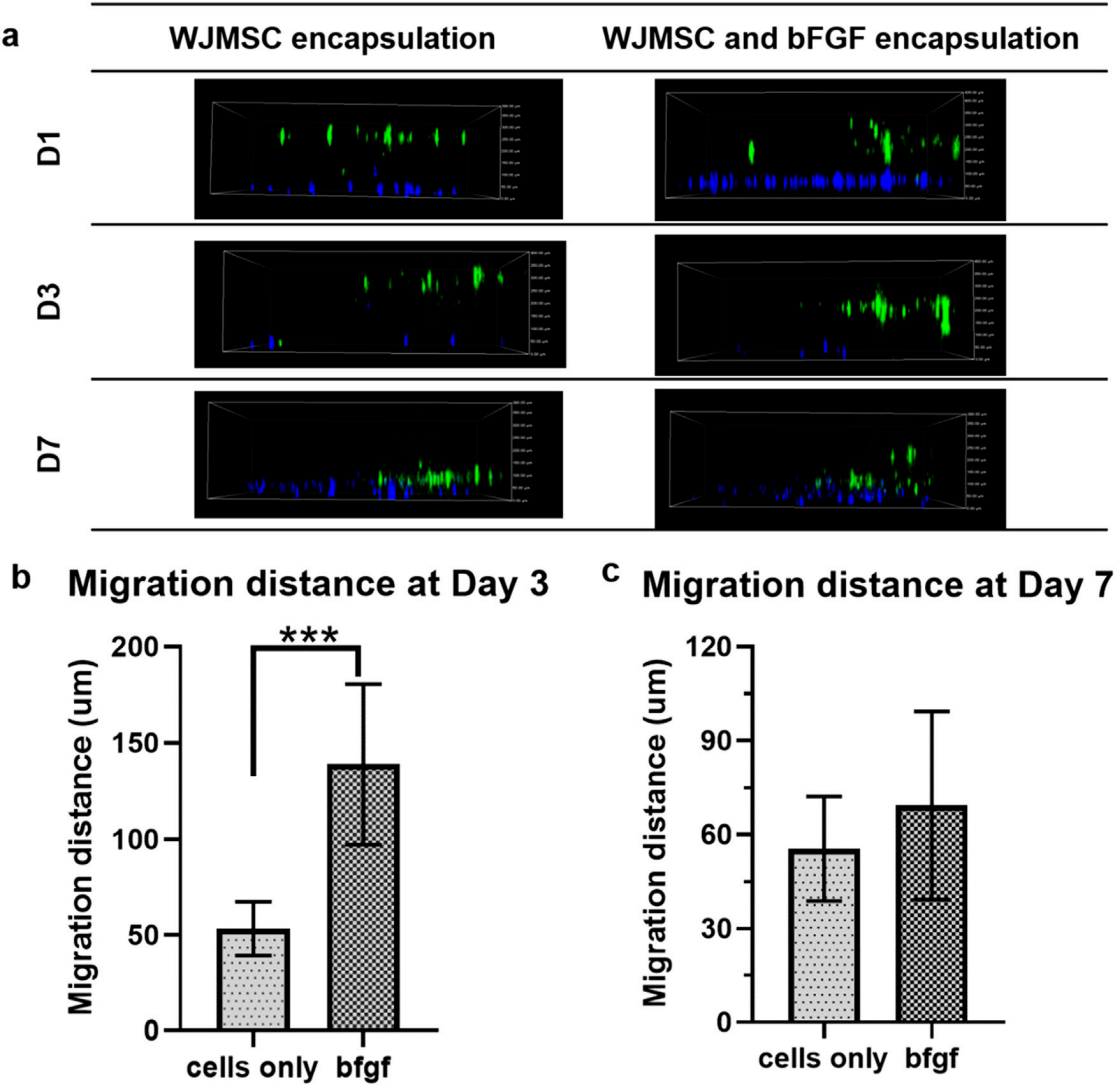
Migration of WJMSCs in genipin-crosslinked gelatin hydrogel with or without bFGF incorporation. The cells were viewed under ×10 magnification and each scale bar represents 100 μm *** indicates p < 0.001. **(A)** 2D view of encapsulated WJMSCs in hydrogel and **(B)** calculated migration distance of encapsulated WJMSCs as compared to previous distance (D1-D3, D3-D7).

Regarding cell migration in genipin-crosslinked gelatin hydrogel, the incorporation of bFGF significantly (p < 0.001) enhanced WJMSCs migration on day three compared to only WJMSCs encapsulation (Figure 9). However, there was no significant difference in migration distance on day seven between WJMSCs encapsulated with or without bFGF incorporation (Figure 9).

#### 3.1.9 Biocompatibility of macrophage

**FIGURE 10:**
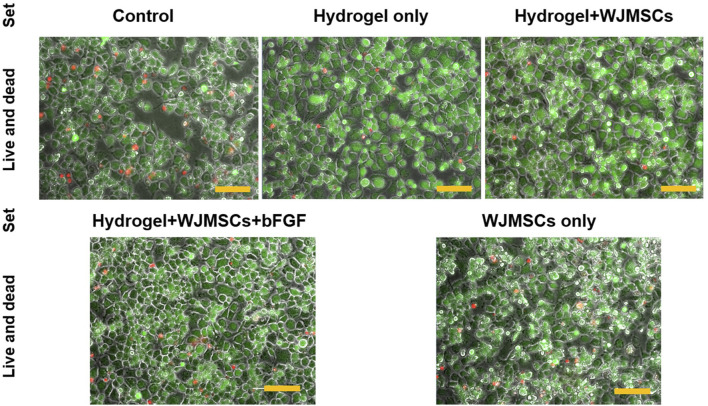
Cellular response via LIVE/DEAD™ Viability/Cytotoxicity assay. Cells were viewed under ×20 magnification and each scale bar represents 100 μm.

After 3 days of co-culture with hydrogel, all sets of macrophages appeared viable, with more greenly-stained cells than redly-stained cells as illustrated in Figure 10. The viability of the macrophages was not affected by encapsulation of WJMSCs, and bFGF incorporation.

#### 3.1.10 Surface marker expression of macrophage

**FIGURE 11:**
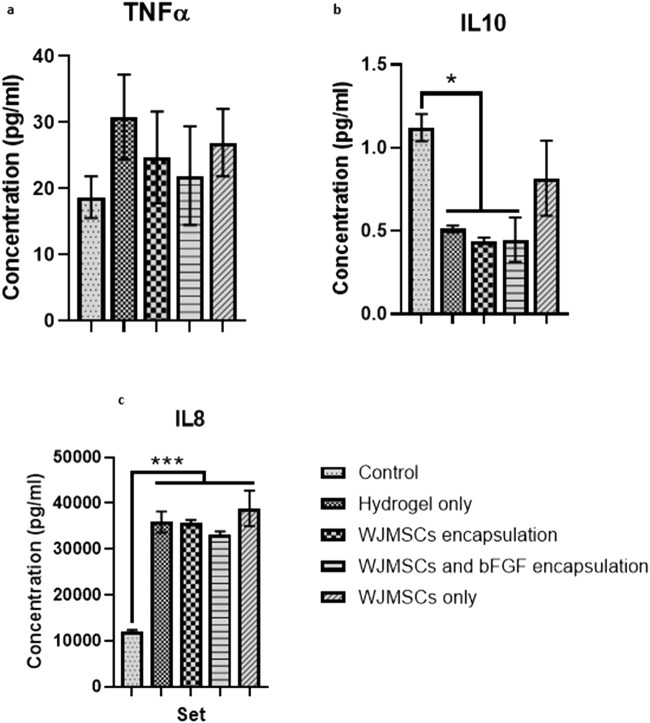
Flow cytometry analysis of macrophage marker expression after 3 days co-culture with WJMSCs in hydrogel. The included markers were **(A)** CD80, **(B)** CD206, **(C)** CD209 and **(D)** CD36 respectively.

The surface marker CD209 expression increased in all groups co-cultured with hydrogel whereas CD36 expression decreased. CD80 and CD206 expression were similar to the control, as depicted in (Figure 11).

#### 3.1.11 Cytokine release of macrophage

**FIGURE 12:**
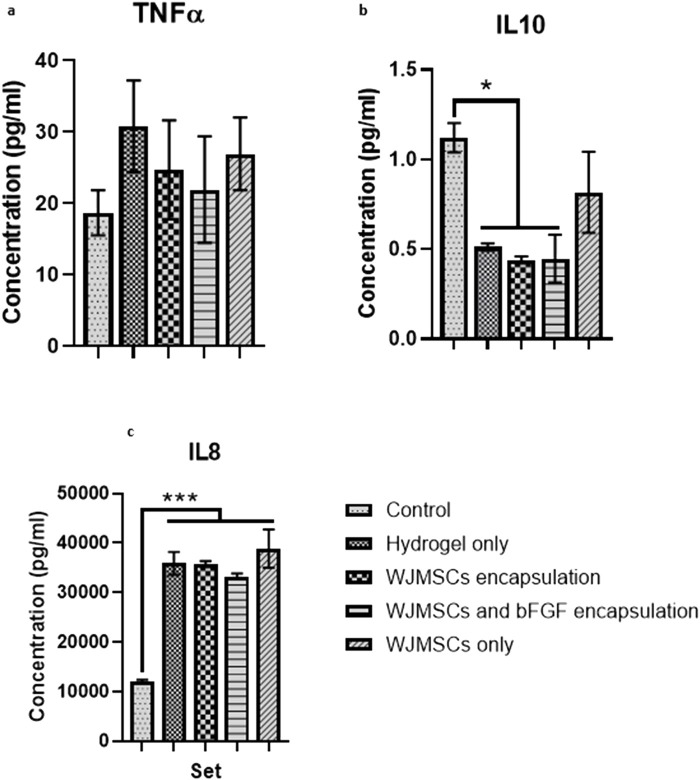
Cytokine release of macrophage. **(A)** TNFα, **(B)** IL-10 and **(C)** IL-8. * indicates p < 0.05, *** indicates p < 0.001.

Co-culture with hydrogel increased TNFα production by macrophages, while incorporation of WJMSCs and bFGF reduced TNFα production, however the differences were insignificant (Figure 12). However, hydrogel co-culture significantly reduced IL-10 production from 1.12 pg/mL (control) to 0.51 pg/mL, 0.44 pg/mL, and 0.45 pg/mL for hydrogel only, hydrogel + WJMSCs, and hydrogel + WJMSCs + bFGF respectively (Figure 12B). Furthermore, hydrogel co-culture with WJMSCs significantly increased IL-8 production from 12,082.7 pg/mL (control) to 35,839.6 pg/mL (hydrogel only), 35,719.9 pg/mL (hydrogel + WJMSCs), 33,281.4 pg/mL (hydrogel + WJMSCs + bFGF) and 38,869.5 pg/mL (WJMSCs only) (Figure 12C).

## 4 Discussion

The initiative of current study is to innovate an in-office hydrogel with regenerative ability in glottic insufficiency. In-office laryngoplasty injection offers several advantages, by preventing invasive procedures and involving only local anesthesia (Bartlett et al., 2012). In current study, the optimised hydrogel offers flexibility, as it can be delivered when it is in liquid form, allows it to accommodate the complex structure of vocal fold. Meanwhile, the crosslinking will be formed *in situ*, allowing it to be adhered to the tissue structure. The crosslinking process is proved to be cell-friendly and growth factor (bFGF) is encapsulated.

Although our previous study proved that the hydrogel formulation (6G 0.4gn and 8G 0.4gn) had favourable physical, chemical and mechanical properties, which demonstrated the potential for cell encapsulation (Ng et al., 2023). Ensuring good cell viability after encapsulation is crucial because the crosslinking process during gelation may have cytotoxic effects, particularly when forming bonds between functional groups (Etter et al., 2018). Genipin’s crosslinking mechanism involves covalent crosslinking, which improves the mechanical strength of the hydrogel while retaining its biodegradable property. This chemical crosslinker does not require additional components to initiate crosslinking, thus reducing the risk of cytotoxic due to by-product during degradation. The current outcome elucidated that 6G 0.4gn and 8G 0.4gn were able to encapsulate and support cell viability, proliferation and cell migration. This might due to the acceptable pore structures of the hydrogel and the ability to transmit nutrients to the encapsulated cells. Pore size is exceptionally important for cell signalling (Qazi et al., 2019). However, 6G 0.4gn exhibited superior cell viability when tested with MTT assay than 8G 0.4gn, possibly due to the dense structure of higher gelatin concentration, leading to less cell viability after 7 days of culturing.

It is worth noting that WJMSCs remained spherical after encapsulation. Cell morphology is closely associated with stiffness, where lower stiffness hydrogels (around 3 kPa) tend to promote cell spreading and elongation, while stiffer hydrogels (around 9 kPa) tend to maintain cell morphology due to pore size variation (Contessi Negrini et al., 2021). Hydrogel’s robustness also influences its degradation kinetics, which will affect cell fate and signalling. Human MSCs have a spherical morphology when encapsulated in a slower degraded matrix (as observed in this study), but exhibit cell communication when degradation occurs (Nicodemus and Bryant, 2008). We observed the hydrogels (6G 0.4gn and 8G 0.4gn) were not fully degraded even after being cultured for 7 days. One of our aims was to develop a hydrogel with slower resorption when injected into the vocal fold. Nevertheless, the inability of hydrogel degradation in the current *in vitro* study may not mimic the exact degradation kinetics in the biological microenvironment. Gelatin is susceptible to proteolytic degradation by collagenase rather than hydrolysis degradation. Vocal fold is mainly built up of collagen, indicating the existence of collagenase, which can help to degrade the hydrogel thus promoting the cell attachment and migration (Tang et al., 2017). This is due to the stiffness of the hydrogel can influence its hydrophobicity and cell recognition receptors (Cui et al., 2021). Therefore, cell viability and cell communication is the main concern of encapsulating cells in a slow degraded and stiffer hydrogel (Luo et al., 2022).

Despite their spherical shape after encapsulation, WJMSCs were observed to be able to migrate through the hydrogel, indicating the presence of attachment ligands that are important for cell adhesion. Gelatin, which is widely used in different applications and is generally regarded as safe by the United States Food and Drug Administration (FDA), contains vital cell communication ligands, such as RGD (Su and Wang, 2015). The cells can only attach on the surface of the hydrogel through RGD-integrin conjugation (Li et al., 2018), which is consistent with the observation in Figure 2. The current formulation, with includes genipin, was able to retain the original structure of gelatin and is compatible with WJMSCs. However, synthetic hydrogels require integration of RGD and N-cadherin to improve cell viability and adhesion of encapsulated BMMSCs (Rong et al., 2020). Despite this, 6G 0.4gn showed better accommodation for WJMSCs encapsulation, as it had a significant improvement in cell viability at day seven. This could be attributed to the concentration of gelatin, which influences the porosity of the hydrogel. Higher gelatin concentration leads to reduced porosity and pore size (Yang et al., 2017). The current study stipulates that the higher concentration of gelatin may result in a denser gelatin structure, causing less porosity and pore size. Additionally, our previous study showed that 8G 0.4gn had a lower pore size distribution than 6G 0.4gn (Ng et al., 2023). It is hypothesised that a higher concentration of gelatin leads to higher viscosity and denser gelatin structure due to higher intermolecular cross bonding of the molecules (Sancakli et al., 2021). More viscous hydrogels require higher injection force (Fan et al., 2021), which might damage the cells’ robustness in long-term culture. This suggests a limitation of this study, as injection force was not measured. Nevertheless, the proliferation of the WJMSCs was supported by the specific staining of ki67, indicating that the cells’ proliferation was maintained up to day seven.

**FIGURE 13:**
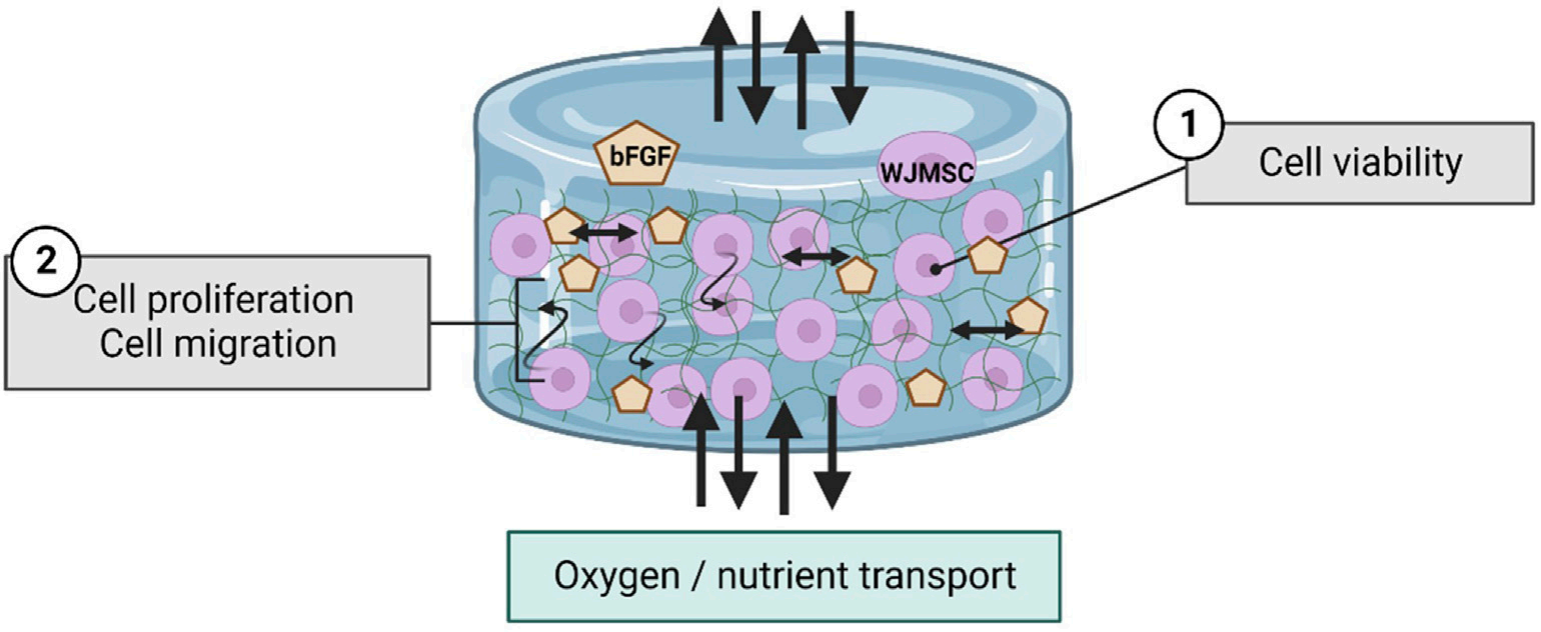
Schematic diagram regarding to the interaction of WJMSCs and bFGF within hydrogel. The figure was created using Biorender.com, the date of access was 08 January 2023.

In this study, 6G 0.4gn exhibited better encapsulation efficiency, led to its selection for bFGF incorporation. The addition of bFGF improved the activity of WJMSCs after 7 days and increased their migration rate in the hydrogel. WJMSCs produce a variety of growth factors, including bFGF. *In vitro* 2D culturing showed that bFGF supplementation enhances the proliferation and differentiation potential of WJMSCs (Panta et al., 2019). bFGF interacts with the upstream genes in the MEK/ERK pathway and regulates the expression of other proteins such as cyclin B1, which promotes proliferation (Chang et al., 2020). The enhanced proliferation of WJMSCs can increase the production of bFGF by the cells, while maintaining their differentiation and immunomodulation ability (Fujimoto et al., 2020). The regenerative ability of WJMSCs is affected by the hypoxia and nutrient conditions of the microenvironment. Previous research suggested that the encapsulated WJMSCs co-culture with injured cells produced less bFGF compared to normal *in vitro* setting (Lech et al., 2020). Thus, the incorporation of bFGF in the current study was deemed advantageous for maintaining the stemness and proliferation of WJMSCs in 3D conditions. bFGF is also vital to modulate the functions of fibroblasts in vocal fold (Yamauchi et al., 2022). The fibroblasts presence in vocal fold is significantly discussed by researchers, which is important in extracellular matrix (ECM) production to retain the viscoelasticity of vocal fold for voice production (Kishimoto et al., 2016). Our genipin-crosslinked gelatin hydrogel is aimed to provide a sustained release of bFGF toward the native tissue to support a long-term regenerative effect, via gradual degradation of the hydrogel matrix (Figure 13). Moreover, the incorporation of bFGF into our hydrogel is relatively direct and easy, with require no additional step.

To prevent adverse events during *in vivo* studies, the immunogenicity of the hydrogel must be studied *in vitro*. In the current study, macrophages co-culture with hydrogel for up to 3 days demonstrated that the hydrogel was non-cytotoxic to macrophages. The expression of CD209, a pattern recognition receptor that affects macrophages phagocytosis ability (Jiang and Sun, 2017), increased after co-cultured with the hydrogel, with or without WJMSCs and bFGF encapsulation. CD209 also initiates innate and adaptive immunity by recognising mannosyl glycans in microorganisms. However, CD209 is found to express in M2-polarised macrophages as well (Buchacher et al., 2015). In contrast, the expression of CD36, a scavenger receptor, expressed by M1-polarised macrophages (Forrester et al., 2018), decreased after co-culture with the hydrogel, with or without WJMSCs and bFGF encapsulation. CD36 recognises foreign material or receptors and promotes phagocytosis by macrophages (Woo et al., 2016). In our study, the expression of CD209 induced by the encapsulation of WJMSCs and bFGF was higher than the control, suggesting a potential anti-inflammatory outcome. This finding aligns with the result by Erndt-Marino et al. (2018), showing that bFGF was able to enhance the anti-inflammatory effects of vocal fold fibroblasts. However, CD80 and CD206 are the most common markers for confirming macrophages polarization into M1 or M2. According to previous research, the polarization of M0 into M1 should express positive CD80 and low CD206, while M2 should express negative CD80 and high CD206 (Bertani et al., 2017), which was not observed in the current study. The insignificant result of CD80 and CD206 in the current study suggests that the macrophages may have remained in M0. However, the M0 state might suggest positive outcome, as it is acknowledged that macrophages play important role in vocal fold regeneration, and over expression of macrophages with myofibroblasts might cause fibrosis and then scarring (Nakamura et al., 2022; Xu and Fan, 2021). It is further justified in our cytokine release test, that with or without encapsulation of WJMSCs and bFGF induced no pro-inflammatory response. Specifically, no insignificant increase in TNFα was observed after co-culture with the hydrogel, with or without WJMSCs and bFGF encapsulation. Although, the hydrogel with WJMSCs and bFGF encapsulation did not demonstrate any anti-inflammatory properties as evidenced by the absence of significant IL10 induction, the significant increase in IL8 secretion after co-culture with the hydrogel, with or without WJMSCs and bFGF, did not justify the pro-inflammation properties of the hydrogel. Previous studies have suggested that IL8 secretion can be induced by both M1 or M2 macrophage, which can lead to pro- and anti-inflammatory effects during innate immunity, respectively (Orekhov et al., 2019; Zheng et al., 2018). Additionally, IL8 can also can be detected in M0 and M2 macrophages (Parisi et al., 2023). Aligned with outcome by (X. Li et al., 2024), the interaction between cells and hydrogel resulted in high secretion of IL8. These findings suggest our hydrogel supports biocompatibility of macrophages that also allows the immunomodulation outcome by the macrophages. Altogether, the outcome may propose the low immunogenicity properties and biocompatibility of the hydrogel, however, the main indicators such as CD80, CD206, TNFα, and IL10 were insignificant. This could be due to the absence of vocal fold fibroblast in the *in vitro* setting, as vocal fold fibroblast plays a vital role in stimulating macrophage polarization (Nakamura et al., 2022).

Based on our previous study, both 6G 0.4gn and 8G 0.4gn showed satisfactory characteristics for vocal fold injection. Both hydrogels exhibited fast gelation time (<20 min), elastic modulus between 2 and 10 kPa, which is more comparable to viscoelasticity of human vocal fold (superior medial: 5.0 kPa; inferior medial: 7.0 kPa) (Chhetri and Rafizadeh, 2014). Following to that, current preliminary study proposed that 6G 0.4gn might be more superior, due to its ability to maintain cellular activities of encapsulated WJMSCs with incorporation of bFGF, while exhibiting low inflammation outcome *in vitro*. In view of these advantages, it is expected to overcome the limitations of existing biomaterials which have no regenerative effect, fast resorption and migration issue (DeFatta et al., 2012; Wang et al., 2020; Wang and Carroll, 2021). However, this outcome needs to be validated in future studies as it has certain limitations that commonly encountered in in vitro studies. For example, the current study only focused on developing a suitable hydrogel for WJMSCs and bFGF encapsulation, and the regenerative ability of the encapsulated WJMSCs and bFGF was not elucidated in this study. In addition, vocal fold fibroblast was not included in this study due to the limited availability of the sample, and this issue will need to be addressed in future studies. The co-culture of the hydrogel with vocal fold fibroblasts is vital to elucidate its regenerative ability by protein expression of vocal fold fibroblasts. Even though, the incorporation of bFGF was proposed to improve the cellular response of WJMSCs, the release sustained of bFGF in the hydrogel remains unknown. The future work should study the release profile of bFGF to demonstrate the hydrogel’s ability to sustain bFGF release both *in vitro* and *in vivo*, considering both short- and long-term effects. Moreover, the gelation time, biodegradation profile and injectability should be re-investigated in an *in vivo* setting due to the physiological and microenvironment difference from *in vitro* work. The positive outcome of the study was emphasised to be a preliminary study, as the *in vitro* study did not represent the real microenvironment such as the culture medium used was not comprised of enzymes and other cells or extracellular matrix in the human vocal fold. Altogether, these factors might influence the degradation kinetics and cellular behaviour of the biomaterials and encapsulated cells (Mazzeo et al., 2019). By referring to Kwon et al. (2021)’s protocol, these can be tested in in vivo, by scoping the vocal fold’s condition, and investigate the intactness of the biomaterial with vocal fold during harvesting.

In ensuring the translational ability of this hydrogel, several aspects should be taken in consideration during the development, such as regulatory approval, supply chain mechanism, quality control of raw materials and feasibility of large-scale manufacturing. The future work needs to further prove the safety and tumorigenesis of current formulation, with incorporation of bFGF and WJMSCs in 6G 0.4gn hydrogel, via several stability tests and *in vivo* model. Moreover, we should also explore the practicality of the fabricated hydrogel. Injectability of the hydrogels were demonstrated in our previous study, showing that the hydrogels were able to be injected after 10 min (approximately time needed by clinician to inject the hydrogel into vocal fold) of polymerisation in room temperature (Ng et al., 2023). It is acknowledged that the injection force was not quantified due to resource limitations; however, the gelation and injection times of the hydrogel are of greater importance due to its time-sensitive nature. Genipin crosslinking should be initiated only when required by the clinician to prevent over-gelation. Therefore, we recommend a customised two-chamber needle to simplify the preparation of gelatin with genipin in a clinical setting. The quality control of WJMSCs should be established by listing out standard protocol starting from the sourcing of umbilical cord, method to extract WJMSCs, culture protocol, characterisation of WJMSCs, cryo-banking, thawing of the cells to the final encapsulation of WJMSCs into the hydrogel. Since the polymerisation of genipin-gelatin is critical to the injection process, large-scale production must be carefully considered when defining the end-product, to ensure long term feasibility and profitability. This poses a potential challenge compared to current biomaterials for vocal fold injection, such as hyaluronic acid and CaHA, which are relatively easy to handle in clinical settings. These limitations and considerations are critical to be contemplated and need to be addressed in future studies.

## 5 Conclusion

The current research proposed a final formulation, 6G 0.4gn that was suitable for WJMSCs encapsulation *in vitro* setting. This formulation supported viability, proliferation and migration of WJMSCs up to 7 days during encapsulation. Moreover, with the incorporation of bFGF into the cell-laden hydrogel, the encapsulated WJMSCs had improved viability and migration. Moreover, 6G 0.4gn also supported biocompatibility of macrophages, which then provided the low immunogenicity effect in in vitro setting. Therefore, this work serves as a proof-of-concept for the firstly introduced genipin-crosslinked gelatin hydrogel which has viscoelasticity, low inflammatory profile and slow biodegradation. These characteristics are deemed to be suitable for vocal fold injection. In addition, WJMSCs and bFGF encapsulation has potential to provide regenerative effect. As the work is done *in vitro* setting, future work should include vocal fold fibroblast to elucidate the regenerative properties, and prove via ex or *in vivo* setting for translational work.

## Data Availability

The original contributions presented in the study are included in the article/supplementary material, further inquiries can be directed to the corresponding author.

## References

[B1] Abbaszadeh H. Ghorbani F. Derakhshani M. Movassaghpour A. A. Yousefi M. Talebi M. (2020). Regenerative potential of wharton’s jelly-derived mesenchymal stem cells: a new horizon of stem cell therapy. J. Cell. Physiology 235 (12), 9230–9240. 10.1002/jcp.29810 32557631

[B2] Aboul-Soud M. A. M. Alzahrani A. J. Mahmoud A. (2021). Induced pluripotent stem cells (iPSCs)-roles in regenerative therapies, disease modelling and drug screening. Cells 10 (9), 2319. 10.3390/cells10092319 34571968 PMC8467501

[B3] Akter F. (2016). “Chapter 2 - principles of tissue engineering,” in Tissue engineering made easy. Editor Akter F. (Academic Press), 3–16.

[B4] Bakhshandeh B. Zarrintaj P. Oftadeh M. O. Keramati F. Fouladiha H. Sohrabi-Jahromi S. (2017). Tissue engineering; strategies, tissues, and biomaterials. Biotechnol. and Genet. Eng. Rev. 33 (2), 144–172. 10.1080/02648725.2018.1430464 29385962

[B5] Bartlett R. S. Thibeault S. L. Prestwich G. D. (2012). Therapeutic potential of gel-based injectables for vocal fold regeneration. Biomed. Mater. Bristol, Engl. 7 (2), 024103. 10.1088/1748-6041/7/2/024103 PMC3369184 22456756

[B6] Bertani F. R. Mozetic P. Fioramonti M. Iuliani M. Ribelli G. Pantano F. (2017). Classification of M1/M2-polarized human macrophages by label-free hyperspectral reflectance confocal microscopy and multivariate analysis. Sci. Rep. 7 (1), 8965. 10.1038/s41598-017-08121-8 28827726 PMC5566322

[B7] Buchacher T. Ohradanova-Repic A. Stockinger H. Fischer M. B. Weber V. (2015). M2 polarization of human macrophages favors survival of the intracellular pathogen Chlamydia pneumoniae. PloS One 10 (11), e0143593. 10.1371/journal.pone.0143593 26606059 PMC4659546

[B8] Chang M.-C. Chen C.-Y. Chang Y.-C. Zhong B.-H. Wang Y.-L. Yeung S.-Y. (2020). Effect of bFGF on the growth and matrix turnover of stem cells from human apical papilla: role of MEK/ERK signaling. J. Formos. Med. Assoc. 119 (11), 1666–1672. 10.1016/j.jfma.2019.12.013 31932202

[B9] Cheng Y.-H. Cheng S.-J. Chen H.-H. Hsu W.-C. (2022). Development of injectable graphene oxide/laponite/gelatin hydrogel containing wharton’s jelly mesenchymal stem cells for treatment of oxidative stress-damaged cardiomyocytes. Colloids Surfaces B Biointerfaces 209, 112150. 10.1016/j.colsurfb.2021.112150 34656814

[B10] Chhetri D. K. Rafizadeh S. (2014). Young’s modulus of canine vocal fold cover layers. J. Voice Official J. Voice Found. 28 (4), 406–410. 10.1016/j.jvoice.2013.12.003 PMC4058419 24491497

[B11] Christodoulou I. Kolisis F. N. Papaevangeliou D. Zoumpourlis V. (2013). Comparative evaluation of human mesenchymal stem cells of fetal (Wharton’s jelly) and adult (Adipose tissue) origin during prolonged *in vitro* expansion: considerations for cytotherapy. Stem Cells Int. 2013, 246134. 10.1155/2013/246134 23533440 PMC3603673

[B12] Contessi Negrini N. Angelova Volponi A. Sharpe P. T. Celiz A. D. (2021). Tunable cross-linking and adhesion of gelatin hydrogels via bioorthogonal click chemistry. ACS Biomaterials Sci. and Eng. 7 (9), 4330–4346. 10.1021/acsbiomaterials.1c00136 34086456

[B13] Corotchi M. C. Popa M. A. Remes A. Sima L. E. Gussi I. Lupu Plesu M. (2013). Isolation method and xeno-free culture conditions influence multipotent differentiation capacity of human wharton’s jelly-derived mesenchymal stem cells. Stem Cell Res. and Ther. 4 (4), 81. 10.1186/scrt232 23845279 PMC3854854

[B14] Cui L. Yao Y. Yim E. K. F. (2021). The effects of surface topography modification on hydrogel properties. Apl. Bioeng. 5 (3), 031509. 10.1063/5.0046076 34368603 PMC8318605

[B15] DeFatta R. A. Chowdhury F. R. Sataloff R. T. (2012). Complications of injection laryngoplasty using calcium hydroxylapatite. J. Voice Official J. Voice Found. 26 (5), 614–618. 10.1016/j.jvoice.2011.08.005 22056892

[B16] Dreiss C. A. (2020). Hydrogel design strategies for drug delivery. Curr. Opin. Colloid and Interface Sci. 48, 1–17. 10.1016/j.cocis.2020.02.001

[B17] Echalier C. Valot L. Martinez J. Mehdi A. Subra G. (2019). Chemical cross-linking methods for cell encapsulation in hydrogels. Mater. Today Commun. 20, 100536. 10.1016/j.mtcomm.2019.05.012

[B18] Erndt-Marino J. Jimenez-Vergara A. C. Diaz-Rodriguez P. Kulwatno J. Diaz-Quiroz J. F. Thibeault S. (2018). *In vitro* evaluation of a basic fibroblast growth factor-containing hydrogel toward vocal fold lamina propria scar treatment. J. Biomed. Mater. Res. Part B, Appl. Biomaterials 106 (3), 1258–1267. 10.1002/jbm.b.33936 PMC5862030 28580765

[B19] Espona-Noguera A. Ciriza J. Cañibano-Hernández A. Orive G. Hernández R. M. Saenz del Burgo L. (2019). Review of advanced hydrogel-based cell encapsulation systems for insulin delivery in type 1 diabetes mellitus. Pharmaceutics 11, 597. 10.3390/pharmaceutics11110597 31726670 PMC6920807

[B20] Etter J. N. Karasinski M. Ware J. Floreani R. A. (2018). Dual-crosslinked homogeneous alginate microspheres for mesenchymal stem cell encapsulation. J. Mater. Sci. Mater. Med. 29 (9), 143. 10.1007/s10856-018-6151-4 30151747

[B21] Fan C. Xu K. Huang Y. Liu S. Wang T. Wang W. (2021). Viscosity and degradation controlled injectable hydrogel for esophageal endoscopic submucosal dissection. Bioact. Mater. 6 (4), 1150–1162. 10.1016/j.bioactmat.2020.09.028 33134608 PMC7588753

[B22] Forrester M. A. Wassall H. J. Hall L. S. Cao H. Wilson H. M. Barker R. N. (2018). Similarities and differences in surface receptor expression by THP-1 monocytes and differentiated macrophages polarized using seven different conditioning regimens. Cell. Immunol. 332, 58–76. 10.1016/j.cellimm.2018.07.008 30077333 PMC7611637

[B23] Fujimoto Y. Yokozeki T. Yokoyama A. Tabata Y. (2020). Basic fibroblast growth factor enhances proliferation and hepatocyte growth factor expression of feline mesenchymal stem cells. Regen. Ther. 15, 10–17. 10.1016/j.reth.2020.03.013 32490062 PMC7256438

[B24] Gärtner A. Pereira T. Armada-da-Silva P. A. S. Amorim I. Gomes R. Ribeiro J. (2012). Use of poly(DL-lactide-ε-caprolactone) membranes and mesenchymal stem cells from the Wharton’s jelly of the umbilical cord for promoting nerve regeneration in axonotmesis: *in vitro* and *in vivo* analysis. Differentiation 84 (5), 355–365. 10.1016/j.diff.2012.10.001 23142731

[B25] Guan N. Liu Z. Zhao Y. Li Q. Wang Y. (2020). Engineered biomaterial strategies for controlling growth factors in tissue engineering. Drug Deliv. 27 (1), 1438–1451. 10.1080/10717544.2020.1831104 33100031 PMC7594870

[B26] Gupta G. Mahajan K. (2024). Acute laryngitis. StatPearls. Treasure Island, Florida: StatPearls Publishing. 30521292

[B27] Hsieh L.-C. Chen C.-K. Chang C.-W. Leu Y.-S. Ho G.-M. (2022). Preliminary clinical outcomes of VOIS-implant in patients with unilateral vocal fold paralysis. Laryngoscope 132 (8), 1622–1629. 10.1002/lary.29958 34817072

[B28] Huang Z. Powell R. Phillips J. B. Haastert-Talini K. (2020). Perspective on schwann cells derived from induced pluripotent stem cells in peripheral nerve tissue engineering. Cells 9 (11), 2497. 10.3390/cells9112497 33213068 PMC7698557

[B29] Jiang S. Sun L. (2017). Tongue sole CD209: a pattern-recognition receptor that binds a broad range of microbes and promotes phagocytosis. Int. J. Mol. Sci. 18 (9), 1848. 10.3390/ijms18091848 28869534 PMC5618497

[B30] Kaboodkhani R. Mehrabani D. Karimi-Busheri F. (2021). Achievements and challenges in transplantation of mesenchymal stem cells in otorhinolaryngology. J. Clin. Med. 10 (13), 2940. 10.3390/jcm10132940 34209041 PMC8267672

[B31] Kishimoto Y. Kishimoto A. O. Ye S. Kendziorski C. Welham N. V. (2016). Modeling fibrosis using fibroblasts isolated from scarred rat vocal folds. Lab. Investig. 96 (7), 807–816. 10.1038/labinvest.2016.43 27111284 PMC4920689

[B32] Koh B. Sulaiman N. Fauzi M. B. Law J. X. Ng M. H. Idrus R. B. H. (2020). Three dimensional microcarrier system in mesenchymal stem cell culture: a systematic review. Cell and Biosci. 10 (1), 75. 10.1186/s13578-020-00438-8 PMC7271456 32518618

[B33] Kwon S. Choi H. Park C. Choi S. Kim E. Kim S. W. (2021). *In vivo* vocal fold augmentation using an injectable polyethylene glycol hydrogel based on click chemistry. Biomaterials Sci. 9 (1), 108–115. 10.1039/d0bm01155j 33244544

[B34] Lasso J. M. Poletti D. Scola B. Gómez-Vilda P. García-Martín A. I. Fernández-Santos M. E. (2018). Injection laryngoplasty using autologous fat enriched with adipose-derived regenerative stem cells: a safe therapeutic option for the functional reconstruction of the glottal gap after unilateral vocal fold paralysis. Stem Cells Int. 2018, 8917913. 10.1155/2018/8917913 29760737 PMC5924970

[B35] Lech W. Sarnowska A. Kuczynska Z. Dabrowski F. Figiel-Dabrowska A. Domanska-Janik K. (2020). Biomimetic microenvironmental preconditioning enhance neuroprotective properties of human mesenchymal stem cells derived from wharton’s jelly (WJ-MSCs). Sci. Rep. 10 (1), 16946. 10.1038/s41598-020-74066-0 33037314 PMC7547118

[B36] Li P. Dou X. Feng C. Schönherr H. (2018). Enhanced cell adhesion on a bio-inspired hierarchically structured polyester modified with gelatin-methacrylate. Biomaterials Sci. 6 (4), 785–792. 10.1039/c7bm00991g 29210373

[B37] Li X. Lin H. Yu Y. Lu Y. He B. Liu M. (2024). *In situ* rapid-formation sprayable hydrogels for challenging tissue injury management. Adv. Mater. 36 (19), 2400310. 10.1002/adma.202400310 38298099

[B38] Luo T. Tan B. Zhu L. Wang Y. Liao J. (2022). A review on the design of hydrogels with different stiffness and their effects on tissue repair. Front. Bioeng. Biotechnol. 10, 817391. 10.3389/fbioe.2022.817391 35145958 PMC8822157

[B39] Mazzeo M. S. Chai T. Daviran M. Schultz K. M. (2019). Characterization of the kinetics and mechanism of degradation of human mesenchymal stem cell-laden poly(ethylene glycol) hydrogels. ACS Appl. Bio Mater. 2 (1), 81–92. 10.1021/acsabm.8b00390 PMC6760666 31555760

[B40] Nakamura R. Bing R. Gartling G. J. Branski R. C. (2022). Macrophages alter inflammatory and fibrotic gene expression in human vocal fold fibroblasts. Exp. Cell Res. 419 (1), 113301. 10.1016/j.yexcr.2022.113301 35931141

[B41] Ng W.-C. Lokanathan Y. Fauzi M. B. Baki M. M. Zainuddin A. A. Phang S. J. (2023). *In vitro* evaluation of genipin-crosslinked gelatin hydrogels for vocal fold injection. Sci. Rep. 13 (1), 5128. 10.1038/s41598-023-32080-y 36991038 PMC10060255

[B42] Nicodemus G. D. Bryant S. J. (2008). Cell encapsulation in biodegradable hydrogels for tissue engineering applications. Tissue Eng. Part B, Rev. 14 (2), 149–165. 10.1089/ten.teb.2007.0332 18498217 PMC2962861

[B43] Orekhov A. N. Orekhova V. A. Nikiforov N. G. Myasoedova V. A. Grechko A. V. Romanenko E. B. (2019). Monocyte differentiation and macrophage polarization. Vessel Plus 3, 10. 10.20517/2574-1209.2019.04

[B44] Panta W. Imsoonthornruksa S. Yoisungnern T. Suksaweang S. Ketudat-Cairns M. Parnpai R. (2019). Enhanced hepatogenic differentiation of human wharton’s jelly–derived mesenchymal stem cells by using three-step protocol. Int. J. Mol. Sci. 20 (12), 3016. 10.3390/ijms20123016 31226809 PMC6627410

[B45] Parisi L. Bianchi M. G. Ghezzi B. Maurizi E. Macaluso G. M. Bussolati O. (2023). Preparation of human primary macrophages to study the polarization from monocyte-derived macrophages to pro- or anti-inflammatory macrophages at biomaterial interface *in vitro* . J. Dent. Sci. 18, 1630–1637. 10.1016/j.jds.2023.01.020 37799917 PMC10547954

[B46] Park S. Kim S. Lim K. Shin Y. Song K. Kang G.-H. (2023). Thermostable basic fibroblast growth factor enhances the production and activity of human wharton’s jelly mesenchymal stem cell-derived extracellular vesicles. Int. J. Mol. Sci. 24, 16460. 10.3390/ijms242216460 38003648 PMC10671285

[B47] Qazi T. H. Tytgat L. Dubruel P. Duda G. N. Van Vlierberghe S. Geissler S. (2019). Extrusion printed scaffolds with varying pore size as modulators of MSC angiogenic paracrine effects. ACS Biomaterials Sci. and Eng. 5 (10), 5348–5358. 10.1021/acsbiomaterials.9b00843 33464076

[B48] Raghav P. K. Mann Z. Ahlawat S. Mohanty S. (2022). Mesenchymal stem cell-based nanoparticles and scaffolds in regenerative medicine. Eur. J. Pharmacol. 918, 174657. 10.1016/j.ejphar.2021.174657 34871557

[B49] Rao L. Qian Y. Khodabukus A. Ribar T. Bursac N. (2018). Engineering human pluripotent stem cells into a functional skeletal muscle tissue. Nat. Commun. 9 (1), 126. 10.1038/s41467-017-02636-4 29317646 PMC5760720

[B50] Rong Y. Zhang Z. He C. Chen X. (2020). Bioactive polypeptide hydrogels modified with RGD and N-cadherin mimetic peptide promote chondrogenic differentiation of bone marrow mesenchymal stem cells. Sci. China Chem. 63 (8), 1100–1111. 10.1007/s11426-020-9772-0

[B51] Sabaghi M. Tavasoli S. Taheri A. Jamali S. N. Faridi Esfanjani A. (2022). Controlling release patterns of the bioactive compound by structural and environmental conditions: a review. J. Food Meas. Charact. 17, 2261–2284. 10.1007/s11694-022-01786-4

[B52] Sancakli A. Basaran B. Arican F. Polat O. (2021). Effects of bovine gelatin viscosity on gelatin-based edible film mechanical, physical and morphological properties. SN Appl. Sci. 3 (1), 8. 10.1007/s42452-020-04076-0

[B53] Sheth S. Barnard E. Hyatt B. Rathinam M. Zustiak S. P. (2019). Predicting drug release from degradable hydrogels using fluorescence correlation spectroscopy and mathematical modeling. Front. Bioeng. Biotechnol. 7, 410. 10.3389/fbioe.2019.00410 31956651 PMC6951421

[B54] Su K. Wang C. (2015). Recent advances in the use of gelatin in biomedical research. Biotechnol. Lett. 37 (11), 2139–2145. 10.1007/s10529-015-1907-0 26160110

[B55] Tang S. S. Mohad V. Gowda M. Thibeault S. L. (2017). Insights into the role of collagen in vocal fold health and disease. J. Voice Official J. Voice Found. 31 (5), 520–527. 10.1016/j.jvoice.2017.01.008 PMC5583023 28359643

[B56] Wang C.-C. Wu S.-H. Tu Y.-K. Lin W.-J. Liu S.-A. (2020). Hyaluronic acid injection laryngoplasty for unilateral vocal fold paralysis-A systematic review and meta-analysis. Cells 9 (11), 2417. 10.3390/cells9112417 33167303 PMC7694408

[B57] Wang T. V. Carroll T. L. (2021). Injection laryngoplasty and novel injectable materials. Curr. Otorhinolaryngol. Rep. 9 (2), 107–112. 10.1007/s40136-021-00331-z

[B58] Woo M.-S. Yang J. Beltran C. Cho S. (2016). Cell surface CD36 protein in monocyte/macrophage contributes to phagocytosis during the resolution phase of ischemic stroke in mice. J. Biol. Chem. 291 (45), 23654–23661. 10.1074/jbc.M116.750018 27646002 PMC5095418

[B59] Wu M. Chen L. Qi Y. Ci H. Mou S. Yang J. (2022). Human umbilical cord mesenchymal stem cell promotes angiogenesis via integrin β1/ERK1/2/HIF-1α/VEGF-A signaling pathway for off-the-shelf breast tissue engineering. Stem Cell Res. and Ther. 13 (1), 99. 10.1186/s13287-022-02770-x 35255978 PMC8900416

[B60] Xu H. Fan G.-K. (2021). The role of cytokines in modulating vocal fold fibrosis: a contemporary review. Laryngoscope 131 (1), 139–145. 10.1002/lary.28507 32293731

[B61] Yamauchi T. Kanazawa T. Hasegawa T. Kurakami K. Konomi U. Hirosaki M. (2022). Long-term results and safety of fibroblast growth factor injection for unilateral vocal fold paralysis. Laryngoscope Investig. Otolaryngol. 7 (3), 799–806. 10.1002/lio2.806 PMC9194994 35734070

[B62] Yang L. Tanabe K. Miura T. Yoshinari M. Takemoto S. Shintani S. (2017). Influence of lyophilization factors and gelatin concentration on pore structures of atelocollagen/gelatin sponge biomaterial. Dent. Mater. J. 36 (4), 429–437. 10.4012/dmj.2016-242 28302946

[B63] Zawani M. Maarof M. Tabata Y. Motta A. Fauzi M. B. (2022). Quercetin-embedded gelastin injectable hydrogel as provisional biotemplate for future cutaneous application: optimization and *in vitro* evaluation. Gels 8 (10), 623. 10.3390/gels8100623 36286124 PMC9601625

[B64] Zheng T. Ma G. Tang M. Li Z. Xu R. (2018). IL-8 secreted from M2 macrophages promoted prostate tumorigenesis via STAT3/MALAT1 pathway. Int. J. Mol. Sci. 20 (1), 98. 10.3390/ijms20010098 30591689 PMC6337597

